# Multilevel lumbar spine infection due to poor dentition in an immunocompetent adult: a case report

**DOI:** 10.1186/s13256-017-1492-z

**Published:** 2017-11-22

**Authors:** Michaela B. Quast, Carrie M. Carr, W. Michael Hooten

**Affiliations:** 1 Department of Anesthesiology, Mayo Clinic Graduate School of Medicine, Rochester, MN USA; 2 Department of Radiology, Mayo Clinic College of Medicine, Rochester, MN USA; 3 Department of Anesthesiology, Mayo Clinic College of Medicine, 200 First St SW, Rochester, MN 55905 USA

**Keywords:** Lumbar spine, Epidural abscess, Dentition, Immunocompetent

## Abstract

**Background:**

Although spinal infections have been reported following dental procedures, development of a spinal infection attributed to poor dentition without a history of a dental procedure in an immunocompetent adult has not been previously reported. Here we provide a case report of a multilevel lumbar spine infection that developed in an immunocompetent adult with poor dentition.

**Case presentation:**

A 63-year-old white male man with past medical history of hypertension presented to a hospital emergency department with a 4-month history of progressively worsening low back pain. A musculoskeletal examination demonstrated diffuse tenderness in his lumbar spine area and the results of a neurological examination were within normal limits. Computed tomography and magnetic resonance imaging of his lumbar spine demonstrated a prevertebral and presacral fluid collection ventral to the L4 to L5 and L5 to S1 interspaces. Blood cultures grew pan-sensitive *Streptococcus intermedius* in four of four bottles within 45 hours. Using computed tomography guidance, three core biopsies of the L4 to L5 interspace were taken and subsequent cultures were positive for *Streptococcus intermedius*. He reported that his last episode of dental care occurred more than 20 years ago and a dental panoramic radiograph demonstrated significant necrotic dentition. Ten teeth were extracted and the necrotic dentition was assumed to be the most likely source of infection. On hospital dismissal, he received a 12-week course of intravenously administered ceftriaxone followed by an 8-week course of orally administered cefadroxil pending repeat imaging.

**Conclusions:**

This case report demonstrates the importance of determining the source of infection in a patient with a spontaneous spinal infection. Even in the absence of a recent dental procedure, dentition should be considered a possible source of infection in an immunocompetent patient who presents with a spontaneous spinal infection.

## Background

Nonsurgery-related spinal infections represent an uncommon but devastating group of infectious diseases. The incidence of spontaneous epidural abscess in a geographically defined population was reported to be 0.88 cases per 100,000 person-years (95% confidence interval, 0.27 to 1.48) [1]. This incidence was comparable to the incidence of spontaneous epidural abscess among hospitalized patients which was reported to be 0.33 to 1.96 abscesses per 10,000 hospital admissions per year [2]. Following use of an epidural catheter for operative anesthesia or postoperative analgesia, the odds of developing an epidural abscess has been reported to range from 0 to 1:1930 [3, 4]. Various spinal infections including lumbar and cervical epidural abscesses [5, 6], discitis [7], facet joint abscess [8], and meningitis [5] have been reported following spinal injections for treatment of spine pain; however, due to heterogeneity in this specialized subgroup of patients, the incidence of spinal infections following spinal injections remains unknown.

Spinal infections, specifically epidural abscesses, most often occur in patients with impaired immune function or a history of an infectious disease [1]; thus, spinal infections are uncommon in immunocompetent individuals. Although spinal infections have been reported following dental procedures [9], development of a spinal infection attributed to poor dentition without a history of a dental procedure in an immunocompetent adult has not been previously reported. Here we provide a case report of a multilevel lumbar spine infection that developed in an immunocompetent adult with poor dentition.

## Case presentation

**Fig. 1:**
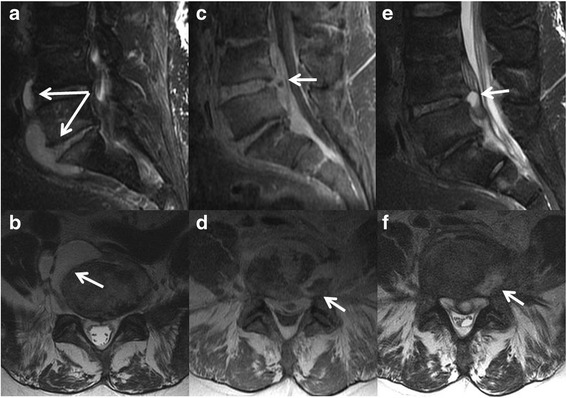
Initial lumbar spine magnetic resonance imaging performed without gadolinium demonstrated extensive abnormal T2 hyperintensity of the L5 interspace (**a** and **b**, sagittal fat-saturated T2-weighted image and axial T2-weighted image at the L5 interspace) indicating discitis. A large multilobulated fluid collection was contiguous with the infected interspace indicating an abscess (*arrow*, **a** and **b**). Follow-up lumbar spine magnetic resonance imaging performed with gadolinium demonstrated extensive abnormal enhancement (**c** and **d**, post-gadolinium sagittal T1-weighted with fat saturation and axial T1-weighted images, respectively) and T2 hyperintensity (**e** and **f**, sagittal T2-weighted with fat saturation and axial T2-weighted at the L4 interspace) involving the L3 to L4, L4 to L5, and L5 to S1 interspaces. There is abnormal paraspinal and epidural enhancing phlegmon with a focal ventral epidural abscess (*arrow*, **c** and **e**) emanating from the L4 to L5 interspace. An additional abscess involving the left posterolateral soft tissues (*arrow*, **d** and **f**) was targeted during biopsy and yielded 10 cc of fluid

A 63-year-old white male man with past medical history of hypertension presented to a hospital emergency department with a 4-month history of progressively worsening low back pain. Immediately prior to seeking emergency care, he reported an episode of fever, rigor, and generalized arthralgia. On initial presentation to the emergency department, his heart rate was 84 beats/minute, blood pressure was 130/65, respiratory rate was 20 breaths/minute, and oral temperature was 38.0 °C. A general examination was significant for a mildly distressed-appearing man and a musculoskeletal examination demonstrated diffuse tenderness in his lumbar spine area to percussive palpation without associated swelling, erythema, or evidence of trauma. The heart, lung, abdominal, and neurological examinations were within normal limits. Laboratory evaluation was significant for a leukocytosis (31,700 × 10^9^/L) with 91% neutrophilia, hypokalemia (2.8 mmol/L), and elevated serum creatinine (1.6 mg/dL). Four sets of blood cultures were obtained from the antecubital area and rapid influenza A and B immunoassays were negative. Urine analysis was negative for nitrates and leukocyte esterase. A chest X-ray showed no evidence of an infectious process in his lungs. Non-contrast magnetic resonance imaging (MRI) of his lumbar spine demonstrated a prevertebral and presacral fluid collection ventral to the L4 to L5 and L5 to S1 interspaces (Fig. 1a and b). The abscess extended into his right psoas musculature. There were findings of discitis involving the L5 to S1 interspace with associated edema of the endplates, although no frank destruction of progressive osteomyelitis was identified. Mild T2 hyperintensity at the L4 to L5 interspace in the presence of a ventral abscess and ventral epidural thickening indicated probable discitis involvement at this level. He was admitted to the hospital with a diagnosis of sepsis and piperacillin/tazobactam was intravenously administered. Blood cultures grew pan-sensitive *Streptococcus intermedius* in four of four bottles within 45 hours. Interventional radiology services were not available at the hospital and he was referred to our tertiary care institution for further evaluation.

**Fig. 2:**
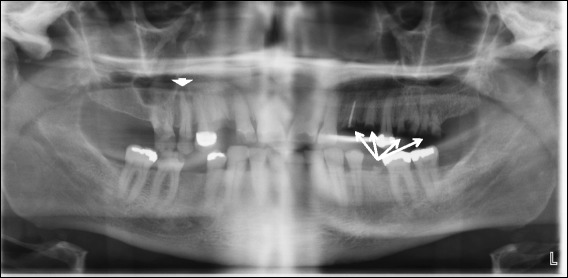
Dental panoramic radiograph demonstrated multiple missing teeth and dental amalgam due to prior dental caries. Most notably there were numerous erosions of the crowns (*arrows*) of many teeth with a mottled appearance of the residual root and periapical lucencies (*arrowhead*). These carious nonrestorable teeth were later extracted

Despite referral, he elected to delay further diagnostic evaluation and treatment for 2 months. On arrival to our tertiary care center, a lumbar spine MRI demonstrated progression of discitis now involving L3 to L4 through L5 to S1 (Fig. 1c–f). There was marked progression of the ventral epidural phlegmon with new development of a 2 cm epidural abscess (Fig. 1c and e; arrowhead). There was progression of mild enhancement of the L4 and L5 vertebral bodies indicative of osteomyelitis. An abscess involving the left side of L4 (Fig. 1c and d) had developed. A computed tomography (CT)-guided biopsy was performed targeting the erosive abscess and 10 cc of fluid was aspirated. In addition, three core biopsies of the L4 to L5 interspace were taken and subsequent cultures were positive for *Streptococcus intermedius*. He had no signs of neurological impairment (that is, normal lower extremity strength and sensation) but a physical examination and dental panoramic radiograph demonstrated significant necrotic dentition (Fig. 2). An examination of his oral cavity was negative for masses, lesions, or ulcerations involving the soft tissues, but several carious nonrestorable teeth were observed that were nontender to palpation or percussion and were nonmobile. He reported that his last episode of dental care occurred more than 20 years ago. As a part of treatment, ten teeth were extracted and the necrotic dentition was assumed to be the most likely source of infection. Other testing included a transesophageal echocardiogram which found no evidence of endocarditis. On hospital dismissal, he completed a 12-week course of ceftriaxone as well as extensive dental work to eradicate the source of infection. He remains on orally administered cefadroxil pending repeat imaging.

## Discussion

Commonly occurring signs and symptoms of an epidural abscess include back pain and focal neurological deficits [1, 9]. However, three aspects of our case report warrant further consideration. First, the organism isolated from L4 to L5 disc space aspirate was a facultative oral bacterium, specifically *Streptococcus intermedius*. More commonly, the organism isolated from epidural abscesses is *Staphylococcus aureus* with typical sources arising from the skin or surgical manipulation of the spine [1, 10].

Second, our patient did not have any known comorbidities, such as diabetes mellitus, history of an infectious disease, or malignancy that could impair immune function. Moreover, he denied use of illicit intravenously administered drugs and there was no history of spinal surgery; both of which have been reported to be risk factors for epidural abscess [1, 10]. Rarely have case reports demonstrated epidural abscess formation from a Streptococcal species in an immunocompetent patient and none have reported progression of the infection to involve the disc space and development of osteomyelitis [11, 12].

Third, oral bacteria have been well documented to spread hematogenously following dental procedures [9]. Unique to our case was that the origin of the spinal infection was necrotic dentition, and there was no history of a recent dental procedure or injury to the oral cavity. However, this does not exclude the potential occurrence of dental manipulation by the patient (that is, use of a toothpick). There have been other case reports that link epidural abscesses to a dental origin [11, 12], but none reported epidural abscesses that have arisen spontaneously in an immunocompetent patient without any recent history of a dental procedure or injury to the oral cavity.

## Conclusions

This case report demonstrates the importance of determining the source of infection in a patient with a spontaneous spinal infection. Even in the absence of a recent dental procedure, dentition should be considered a possible source of infection in an immunocompetent patient who presents with a spontaneous spinal infection.

## Abbreviations

CT: Computed tomography
MRI: Magnetic resonance imaging
